# Mental health and social difficulties of late‐diagnosed autistic children, across childhood and adolescence

**DOI:** 10.1111/jcpp.13587

**Published:** 2022-02-16

**Authors:** Will Mandy, Emily Midouhas, Mariko Hosozawa, Noriko Cable, Amanda Sacker, Eirini Flouri

**Affiliations:** ^1^ Research Department of Clinical, Educational and Health Psychology UCL London UK; ^2^ Department of Psychology and Human Development Institute of Education UCL London UK; ^3^ Department of Epidemiology and Public Health UCL London UK; ^4^ Institute for Global Health Policy Research National Center for Global Health and Medicine Tokyo Japan; ^5^ 12847 Department of Paediatrics and Adolescent Medicine Juntendo University Tokyo Japan

**Keywords:** Autism Spectrum Disorder, diagnosis, co‐occurring mental health conditions, trajectories, Millennium Cohort Study

## Abstract

**Background:**

Autism can be diagnosed from 2 years of age, although most autistic people receive their diagnosis later than this after they have started education. Research is required to understand why some autistic children are diagnosed late, and the level and nature of unmet need prior to diagnosis for late‐diagnosed children.

**Methods:**

We examined trajectories of emotional, behavioural and social difficulties (EBSDs) across childhood and adolescence, comparing ‘earlier‐diagnosed’ (diagnosed 7 years or younger) with ‘late‐diagnosed’ (diagnosed between 8 and 14 years) autistic children. Data were from the Millennium Cohort Study, a population‐based UK birth cohort. EBSDs were measured using the parent‐report Strengths and Difficulties Questionnaire, at 3, 5, 7, 11 and 14 years. We used Growth Curve Modelling to investigate levels and rates of change in these difficulties, and to compare earlier‐ (*n* = 146) and late‐diagnosed (*n* = 284) autistic children.

**Results:**

Aged 5, earlier‐diagnosed autistic children had more emotional (i.e., internalising), conduct, hyperactivity and social difficulties; although clinical difficulties in these areas were nevertheless common in late‐diagnosed children. There was a faster annual increase in scores for all domains for late‐diagnosed children, and by age 14 years, they had higher levels of EBSDs. These results persisted when we ran adjusted models, to account for the late‐diagnosed group having higher rates of late‐diagnosed attention deficit/hyperactivity disorder, higher IQ, a higher proportion of females and older and more educated mothers.

**Conclusions:**

Emotional, behavioural and social difficulties are associated with, and may influence, the timing of autism diagnosis. Late‐diagnosed autistic children often have high levels of mental health and social difficulties prior to their autism diagnosis, and tend to develop even more severe problems as they enter adolescence.

## Introduction

Autism spectrum disorder (hereafter ‘autism’) is a neurodevelopmental condition that, based on current conventions, is diagnosed in 1%–2% of the population (e.g., Centers for Disease Control & Prevention, 2016). It is characterised by difficulties with communication, social interaction, flexibility and sensory processing (American Psychiatric Association, 2013). Autism is usually accompanied by emotional, behavioural and neurodevelopmental difficulties (Lai et al., 2019). Most autistic children (70%) have at least one diagnosable mental health condition, and many (41%) have two or more (Simonoff et al., 2008).

Whilst autism can be reliably diagnosed by expert clinicians in 2‐year‐olds, in practice most people are formally identified later than this (Daniels & Mandell, 2014). There is considerable variability in the age at which people receive an autism diagnosis. Many are only diagnosed once they start primary school; others are not picked up until adolescence; and some reach adulthood with their autism unrecognised (Brugha et al., 2011; Hosozawa, Sacker, Mandy, et al., 2020).

As the goal of timely autism diagnosis has been pursued in many health systems, a growing body of research has sought to describe the characteristics of later‐diagnosed autistic children (Daniels & Mandell, 2014). Such work has the aim of identifying risk factors for late diagnosis that can be used to modify practice, and can show the extent to which a late diagnosis reflects a period in which a child had clinical needs that were overlooked.

The extant literature has tended to define late‐diagnosed children as those not identified as having autism until after they started primary education (e.g., Jónsdóttir, Saemundsen, Antonsdóttir, Sigurdardóttir, & Ólason, 2011; Miodovnik, Harstad, Sideridis, & Huntington, 2015). Compared to those who received a timely diagnosis, such late‐diagnosed autistic children tend to: (a) have less severe autistic symptoms (Sheldrick, Maye, & Carter, 2017); (b) show higher IQ and better language development (Jónsdóttir et al., 2011); (c) be female (Shattuck et al., 2009); (d) have parents who are less educated (Bickel, Bridgemohan, Sideridis, & Huntington, 2015) and (e) be non‐white (Daniels & Mandell, 2014; Shattuck et al., 2009).

In addition, there is some evidence that the emotional, behavioural and social difficulties (EBSDs) that commonly co‐occur with autism can impact upon diagnostic timing (Daniels & Mandell, 2014). In qualitative research, the reports of late‐diagnosed autistic people suggest that diagnostic overshadowing can occur, whereby emotional (i.e., internalising) and behavioural (e.g., attention‐deficit/hyperactivity disorder [ADHD], conduct problems) difficulties masked their underlying autism from professionals, delaying its recognition (Bargiela, Steward, & Mandy, 2016). This idea has been supported with respect to ADHD by several quantitative studies, which show that a prior ADHD diagnosis is associated with later recognition of autism (e.g., Miodovnik et al., 2015).

Whilst there is consistent evidence that an ADHD diagnosis is associated with a delayed autism diagnosis, it is currently unclear whether other EBSDs that commonly co‐occur with autism (e.g., internalising problems, conduct problems and peer relationship problems) impact on the timing of diagnosis. The literature to date has relied mainly on reports of prior nonautistic diagnoses received from professionals, rather than direct symptom measurement (Daniels & Mandell, 2014). Such approaches overlook co‐occurring difficulties that, for one reason or another, have not attracted a formal diagnosis. One recent Australian study addressed this limitation by investigating timing and stability of ASD diagnosis in relation to the trajectory of prospectively measured EBSDs in two cohorts of children (Birth Cohort, aged 6–12 years, *N* = 66; Kinder Cohort, aged 10–16 years, *N* = 77) (May et al., 2021). Using a single measure of overall EBSDs, they found distinct trajectories for earlier‐diagnosed and later‐diagnosed groups. In both cohorts, a similar pattern of findings emerged: the earlier‐diagnosed group initially had the highest levels of EBSDs, but these declined over time; whereas the later‐diagnosed group started with lower levels of EBSDs, but these increased during the study period.

In the current study, we use longitudinal data from the UK’s Millennium Cohort Study to chart trajectories of EBSDs of autistic children from early childhood to middle adolescence (ages 3–14 years). We seek to replicate and extend the findings of May et al. (2021) in a UK sample, leveraging our access to a larger sample size by: (i) investigating subtypes of EBSD, rather than treating this as a unitary construct, to accommodate the potential for distinct trajectories for different types of EBSD; (ii) extending the age range studied to include EBSDs reported at aged 3 and 5 years and (iii) using statistical methods that can account for both between‐ and within‐child variation in ESBDs. We will investigate whether there are patterns of within‐person change characteristic of late‐diagnosed autistic children, who we define as those receiving their diagnosis after the early stages of their primary education (i.e., after age 7 years). These analyses, drawing on prospective data from a nationally representative sample of children, can increase understanding of why some are diagnosed with autism late. Also, as they map maladaptation from early childhood, they will provide insights into the level and nature of clinical need in those with late‐diagnosed autism, prior to diagnosis.

## Methods

### Participants and procedure

The Millennium Cohort Study (MCS; www.cls.ioe.ac.uk/mcs) is a longitudinal survey of 19,243 families drawing its sample from all births in the United Kingdom over a year, beginning in September 2000. Ethical approval was gained from NHS Multi‐Centre Ethics Committees, and parents gave informed consent before interviews took place. We used data from data collection rounds (‘sweeps’) 2–6 (at around 3, 5, 7, 11 and 14 years, respectively), when emotional, social and behavioural difficulties were measured in MCS.

In sweeps 3, 4, 5 and 6, the main parent was asked: ‘Have you ever been told that your child has Autism, Asperger's syndrome or autistic spectrum disorder?’ Using records for only one child per family (the first‐born for multiparous births), our analytic sample (*N* = 430) comprised children whose main parent‐reported ‘yes’ to this question in at least one of sweeps 3, 4, 5 and 6, who had valid data on this question in at least three out of four sweeps and who had a stable diagnosis. We conducted a bias analysis comparing our analytic sample with those whose parents answered ‘yes’ to the above question in at least one wave but who were excluded from the analytic sample (*n* = 148). There were no significant differences between the two samples in any of the background variables (Table S1).

### Measures

*Age of diagnosis* was measured via parent report of whether they have been told that their child has Autism, Asperger’s or autism spectrum disorder, grouped into two categories: (a) an ‘earlier‐diagnosed group’, in which parents first reported their child had an autism diagnosis by age 7 (*n* = 146) and (b) a ‘late‐diagnosed group’, where parents first reported their child had an autism diagnosis between ages 8 and 14 (*n* = 284).

*Child emotional, behavioural and social difficulties* were measured at ages 3, 5, 7, 11 and 14 years with the parent‐reported Strengths and Difficulties Questionnaire (SDQ), which has established reliability and validity (Goodman, 1997) and has been extensively used with autistic children (e.g., Colvert et al., 2021). Scales from the four domains of difficulties measured by the SDQ were used: hyperactivity (e.g., ‘Constantly fidgeting or squirming’), emotional problems (e.g., ‘Nervous or clingy in new situations, easily loses confidence’), conduct problems (e.g., ‘Often has temper tantrums or hot tempers’) and peer problems (e.g., ‘Picked on or bullied by other children’).

We also describe the child and family background characteristics of the autism sample at baseline (age 5 years when information about autism diagnosis was first obtained in MCS) by diagnostic age. Child factors were age in years, gender (female compared to male), ethnicity (white compared to other ethnicity), age of parent‐reported diagnosis of ADHD and general cognitive ability at age 5. Age of ADHD diagnosis was measured as a confounding variable given the elevated risk of co‐occurring ADHD amongst autistic individuals (Reiersen, Constantino, Volk, & Todd, 2007; Simonoff et al., 2008; Steinhausen et al., 2006). In our autistic sample, about 30% reported a diagnosis of ADHD. The diagnosis was based on the primary caregiver’s answer to the question, ‘Has a doctor or health professional ever told you that [Cohort child’s name] had ADHD?’ at any wave (across ages 5–14). A variable was created with three categories: (a) Received an ADHD diagnosis by age 7, (b) Received an ADHD diagnosis between ages 8 and 14, and (c) Did not receive an ADHD diagnosis. In line with previous MCS research, the general intellectual ability was indexed with a factor score derived from principal components analysis of age‐adjusted scores from three ability assessment tests: the BAS Naming Vocabulary scale, the BAS Pattern Construction scale (spatial problem solving) and the BAS Picture Similarities scale (nonverbal reasoning) (see Hanscombe et al., 2012).

Family factors were maternal education (mother had a university degree or not by age 5), mother’s age at the birth of the child (measured in exact age in years) and family socioeconomic disadvantage (SED). Family SED was measured at age 5 by taking the mean of four binary items indexing economic and material deprivation: overcrowding (>1.5 people per room excluding bathroom and kitchen), not owning the home, receipt of means‐tested income supportand income poverty (measured as having a family income less than 60% of the median household income in the United Kingdom) (Malmberg & Flouri, 2011).

### Data analytic plan

Initially, we ran bivariate statistical tests comparing the earlier‐diagnosed and late‐diagnosed groups on the aforementioned child and family factors. We used design‐based (to account for the MCS sampling strategy) Pearson chi‐square tests of association for nominal variables and independent samples *t*‐tests for continuous variables. To examine the patterns of EBSDs over time for autistic children by age of diagnosis, we fitted two‐level growth curve models, with repeated measures of SDQ difficulties scores at ages 3, 5, 7, 11 and 14 (Level 1) nested within children (Level 2). To capture individual trajectories, these models have a random intercept and slope for age. We fitted two models for each SDQ domain. In Model 1, both age and age‐squared were included to capture the non‐linear trajectories of difficulties, allowing them to vary between children. Age was centred at age 5, the first point at which we had information on autism diagnosis. We specified a fixed effect of age of diagnosis at the intercept (age 5) and interaction of age of diagnosis and time‐varying age on the instantaneous linear rate of change in difficulties (‘autism diagnosis between 8 and 14 by age’). In Model 2, to assess whether the associations between age of diagnosis and child difficulties were robust to possible confounding, we adjusted for those child and family background factors that were found to be significantly different at the *p* < .20 level between earlier‐diagnosed and late‐diagnosed children in the first part of the analysis (age of ADHD diagnosis, general cognitive ability, gender, maternal education and age of mother’s first birth). We also specified interaction terms with age for each of these three factors as we did for the age of diagnosis variable, to assess the effects of these variables on the annual rate of change in difficulties, enabling us to capture longitudinal relationships in addition to the cross‐sectional associations with difficulties at age 5. For our dependent variables (SDQ scores), we did not have missing data in the models given multilevel models allow for unbalanced data and therefore retain cases with at least one valid response across waves. However, because of some missing data for our study variables in the analytic sample (1–16% of values were missing across covariates), we multiply imputed missing data. We generated 25 imputed datasets (Graham et al., 2007) in SPSS20 using the Markov Chain Monte Carlo procedure. In the imputation model, we included all of the covariates as a predictor and predicted variables. We fitted our models in Stata17 using the MI (multiple imputations) estimate command which performs individual analyses for each of the imputed datasets, collects estimates of coefficients and their variance–covariance estimates, applies Rubin’s combination rules (Rubin, 1996) to the collected estimates, and reports pooled results. Therefore, we had a sample size of 430 in both Model 1 and Model 2. Model estimates were adjusted for the area stratum to reflect the sampling design of MCS.

## Results

The majority of our sample (*n* = 284, 67%) were in the late‐diagnosed group, with a reported autism diagnosis between ages 8 and 14 years. Only 33% (*n* = 146) had a parent‐reported autism diagnosis by age 7. Table 1 presents participant characteristics by age of diagnosis. Compared to the earlier‐diagnosed group, children in the late‐diagnosed group were more likely to be female and to have a higher general cognitive ability at age 5; and were more likely to be late‐diagnosed with ADHD if they receive a late ASD diagnosis (and more likely to be early‐diagnosed with ADHD if they received an early ASD diagnosis).

**Table 1:** Child, parent and family factors by age of autism diagnosis

Categorical variables	Diagnosis by age 7		Diagnosis between 8 and 14		
	Freq.	%	Freq.	%	*F* ^a^
Female child	19	11.70	76	27.70	9.66**
Child has white ethnicity	127	89.19	256	92.79	1.58
ADHD diagnosis					
No diagnosis	101	72.50	191	69.63	5.380**
Late diagnosis	16	9.35	63	21.13	
Early diagnosis	29	18.15	30	0.24	
Mother has university degree (by age 14)	25	15.62	54	22.75	0.14

Continuous variables	*n* (valid)	Mean (*SE*)	*n* (valid)	Mean (*SE*)	*t* ^b^
Family socioeconomic disadvantage (age 5)	143	0.24 (0.03)	276	0.25 (0.02)	−0.14
Mother’s age at birth of child	146	28.61 (0.49)	280	27.83 (0.42)	0.65
Child general cognitive ability (age 5)	99	91.92 (1.96)	262	95.67 (1.10)	−2.82****

Ns are unweighted.

^a^ *F* statistic for design‐based Pearson chi‐square that is converted to *F* test to account for the MCS sampling design.

^b^ *t*‐test statistic.

** *p* < .01; ***p < .001. Means and percentages are weighted.

### Growth curve modelling

Descriptive statistics of the mean SDQ scores at ages 3, 5, 7, 11 and 14 years, by group, are presented in Table S2. Additionally, Table S3 shows the proportion of children at each time point with scores in the ‘borderline abnormal’ and ‘abnormal’ ranges of the SDQ, by age of diagnosis.

#### Unadjusted model (Model 1)

Late‐diagnosed children had statistically significantly fewer emotional symptoms (*b* = −0.610, *p* < .01), conduct problems (*b =* −0.438, *p* < .01) hyperactivity (*b* = −1.331, *p* < .001) and peer problems (*b* = −1.410, *p* < .001) around age 5, compared to children diagnosed by age 7. The full regression results are in Tables S4–S7.

Moreover, there was a faster annual increase in scores for all domains for late‐diagnosed children, relative to those with an earlier diagnosis, shown by the significant ‘late diagnosis x age’ coefficients (emotional problems: *b* = 0.084, *p* < .01; conduct problems: *b* = 0.109, *p* < .001; hyperactivity: *b* = 0.111, *p* < .001 and peer problems: *b* = 0.135, *p* < .001; Tables S4–S7).

#### Adjusted model (Model 2)

For each SDQ domain, adjustment for ADHD status, cognitive ability, female gender, mother’s education and mother’s age of first birth resulted in no changes to the significance of the effects of age of diagnosis on scores at age 5 or on the rate of change in difficulties over time (Tables S4 to S7). However, the sizes of coefficients were reduced for the effect of a late diagnosis at age 5 (emotional problems: *b* = −0.507, *p* < .01, conduct problems: *b* = −0.359, *p* < .05; hyperactivity: *b* = −1.149, *p* < .001; peer problems: *b* = −1.357, *p* < .001) and on the rate of change in problems (emotional problems: *b* = 0.076, *p* < .01; conduct problems: *b* = 0.099, *p* < .001; hyperactivity: *b* = 0.111, *p* < .001; peer problems: *b* = 0.130, *p* < .001) compared to Model 1, suggesting that these individual factors partly explain the differences in difficulties for children who were diagnosed late compared to by age 7.

**Figure 1:**
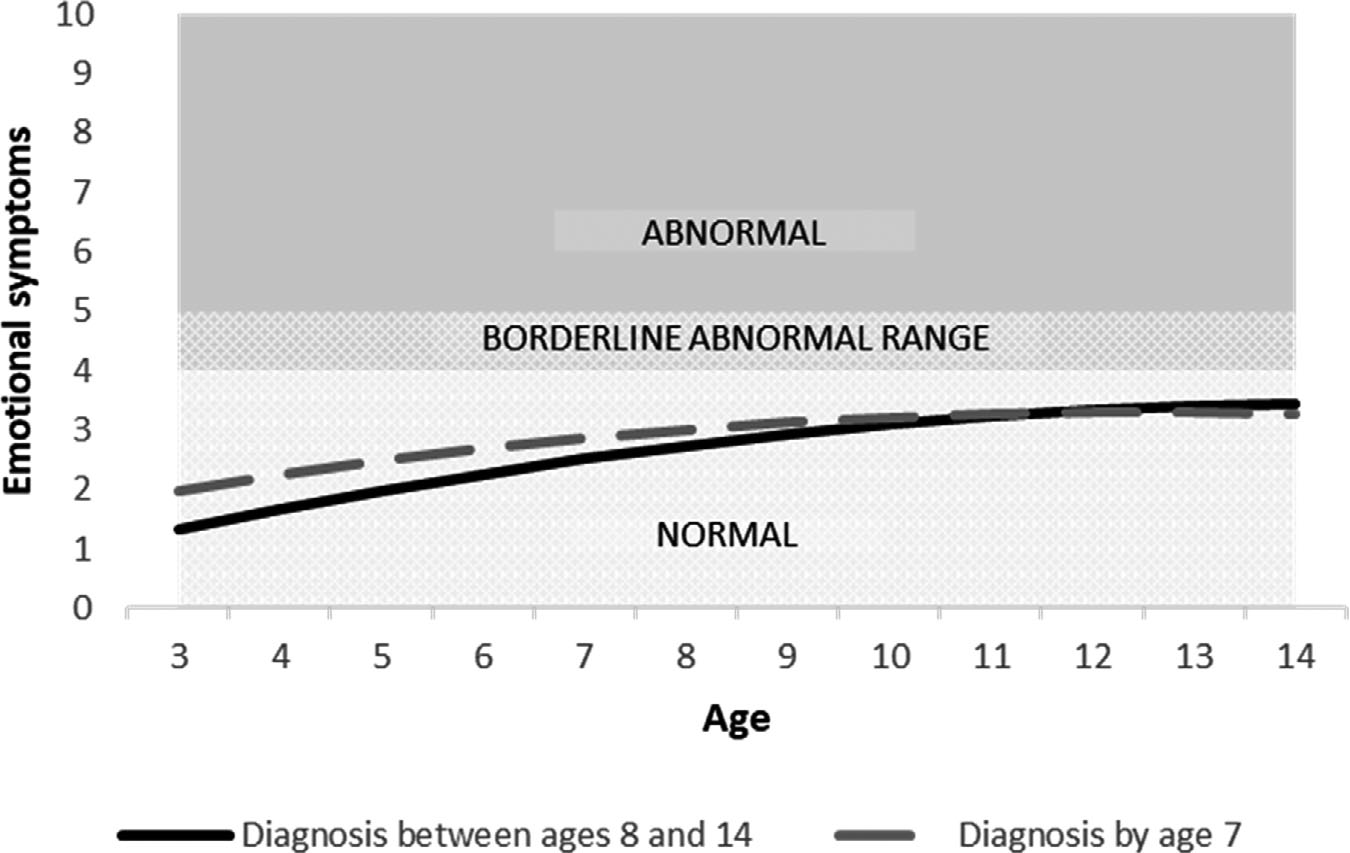
Predicted emotional symptom trajectories by age of diagnosis (Model 2, adjusted model)

**Figure 2:**
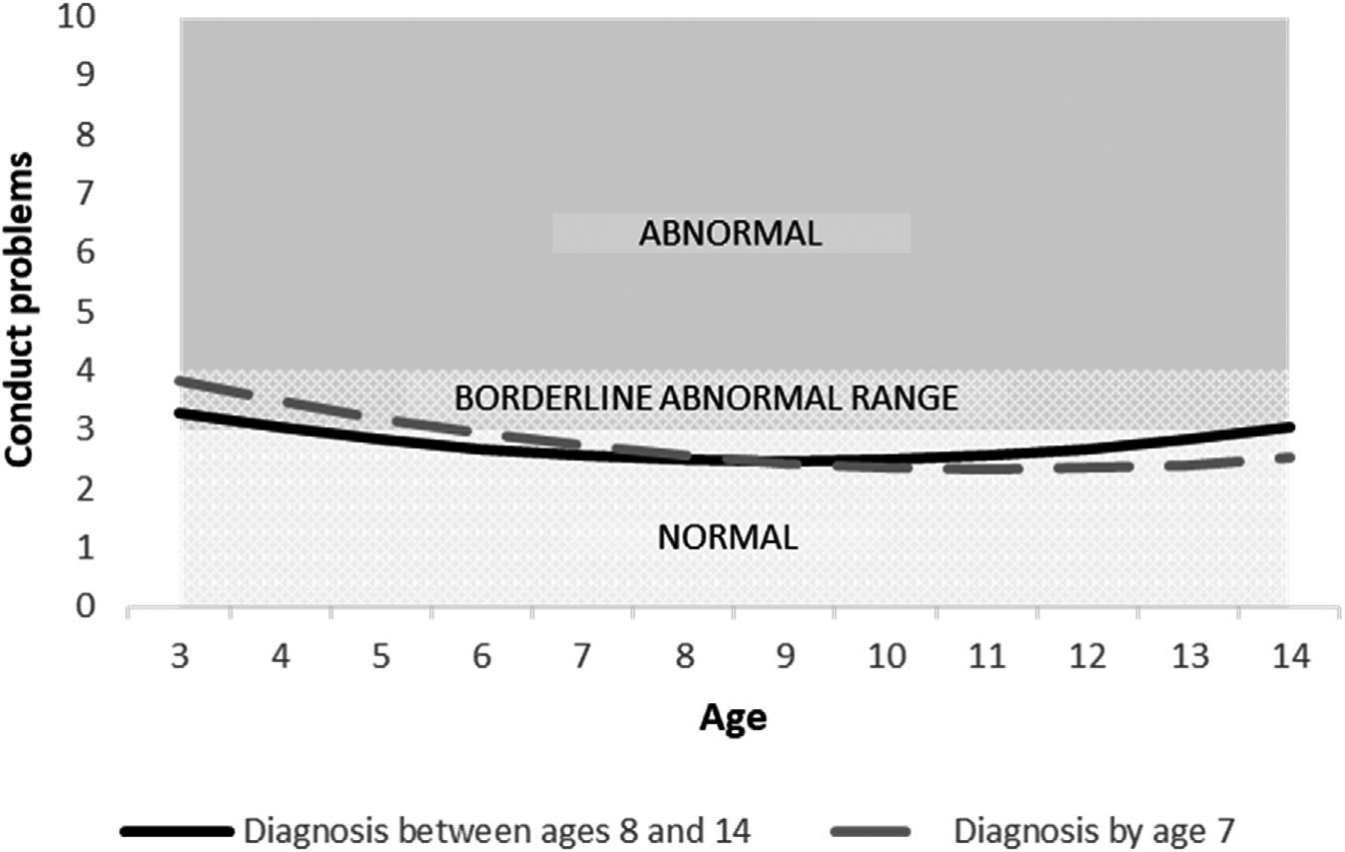
Predicted conduct problems trajectories by age of diagnosis (Model 2, adjusted model)

**Figure 3:**
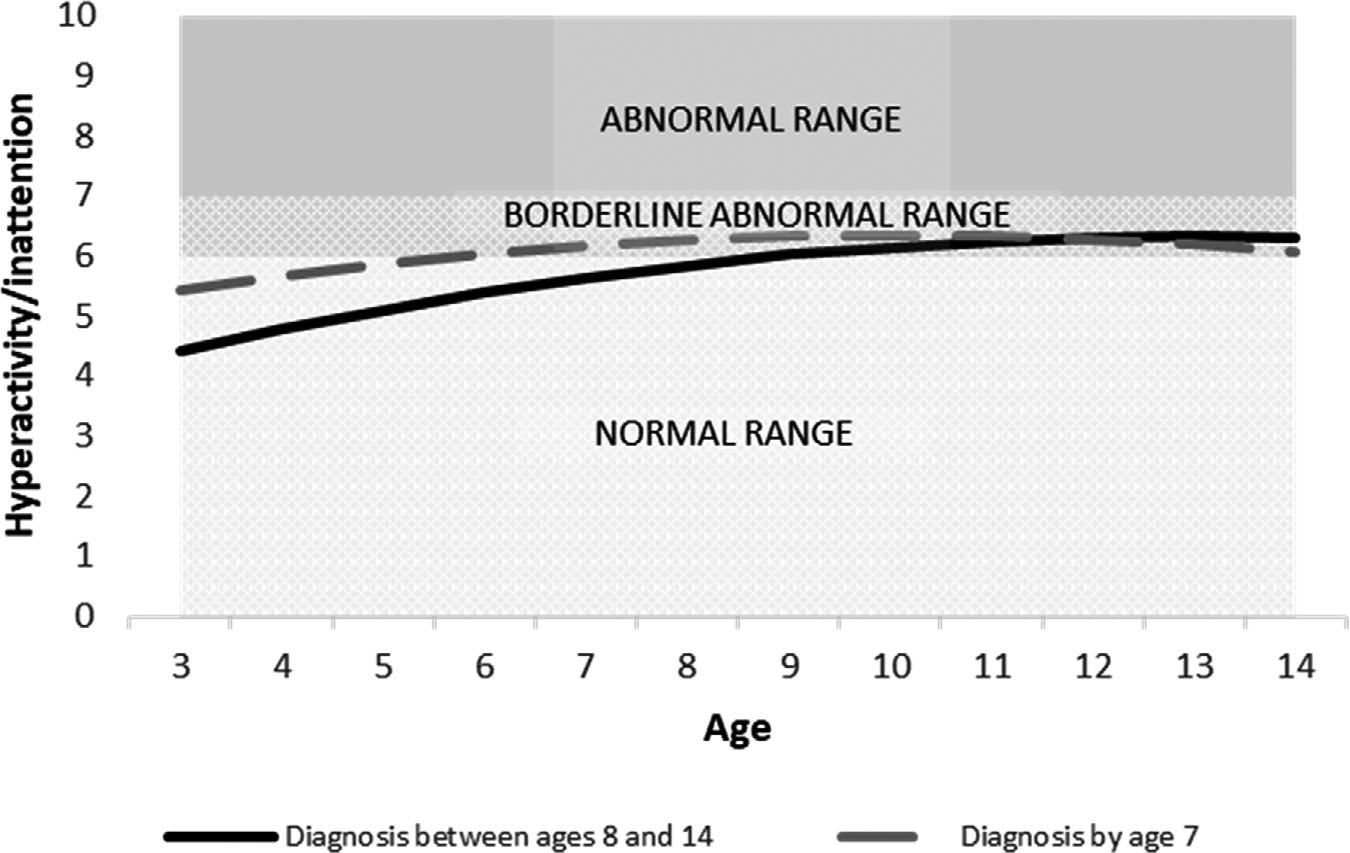
Predicted hyperactivity/inattention trajectories by age of diagnosis (Model 2, adjusted model)

**Figure 4:**
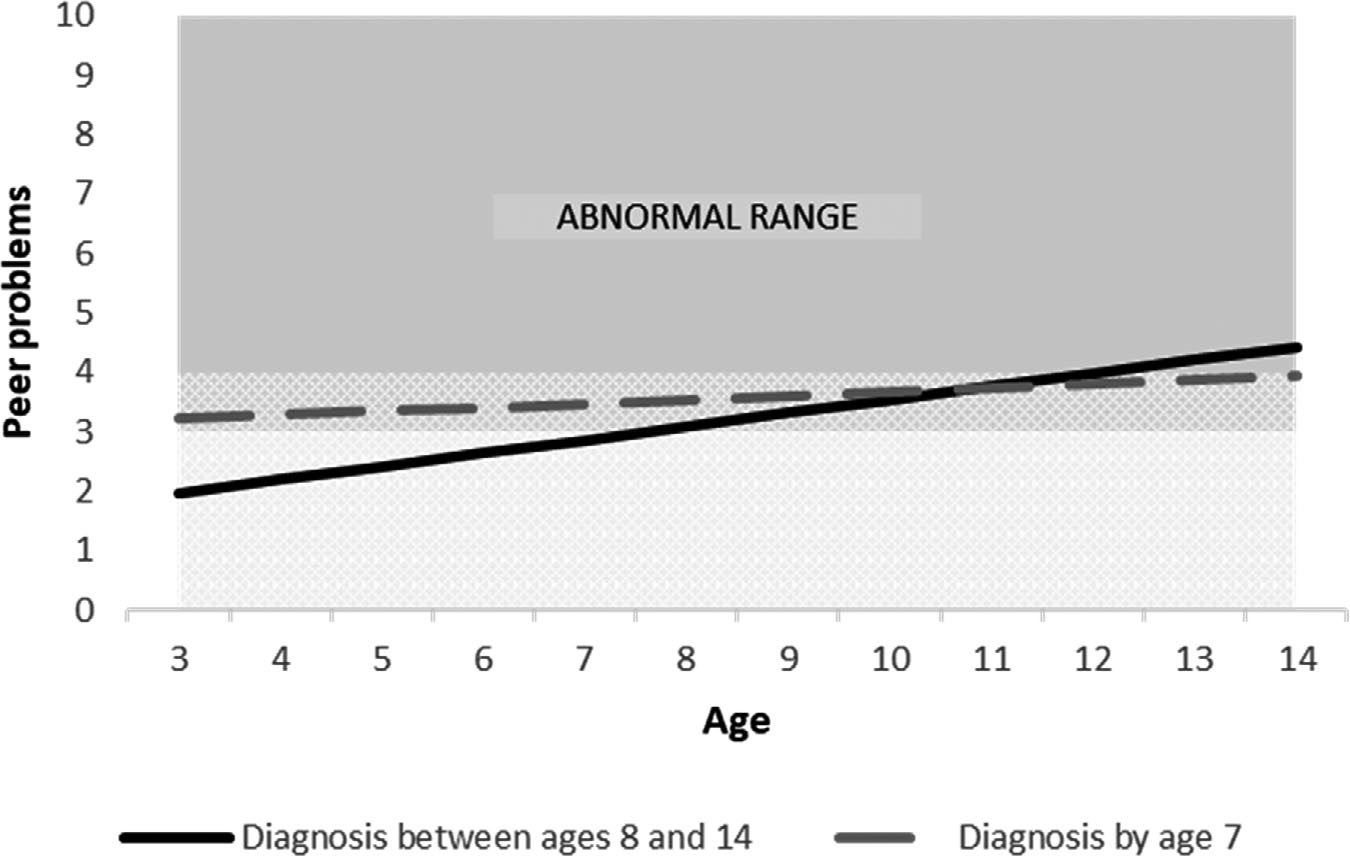
Predicted peer problems trajectories by age of diagnosis (Model 2, adjusted model)

To illustrate the findings from these growth curve models, and specifically to unpack the interactions between the age of first diagnosis and age, we plotted the predicted trajectories for illustrative cases representing our two groups (earlier‐diagnosed and late‐diagnosed). These cases are plotted for all reference groups of categorical variables (boys, mothers without a university degree, early ADHD diagnosis) and the mean of each continuous variable (IQ, age of mother’s first birth). These plots are shown, for the adjusted models, in Figures 1, 2, 3, 4.

## Discussion

We conducted a prospective, population‐based study to compare trajectories of EBSDs of autistic children depending on when they received their autism diagnosis. By contrasting those diagnosed with autism by age 7 (‘earlier diagnosis’) with those diagnosed aged 8–14 years (‘late diagnosis’), we sought to learn more about late‐diagnosed children, to understand their needs better and to inform efforts to improve timely recognition of autism. In line with previous research, compared to earlier‐diagnosed children, those diagnosed after 7 years of age had higher IQ (Shattuck et al., 2009) and were more likely to be female (Brett, Warnell, McConachie, & Parr, 2016). Earlier‐diagnosed and late‐diagnosed children had distinct patterns of EBSDs over time. Compared to earlier‐diagnosed children, the late‐diagnosed group tended to have milder problems in early childhood but then showed a steeper growth in their difficulties, such that by the end of our study period they had higher levels of emotional and behavioural problems and more social difficulties.

Our findings contradict those of previous studies that suggested diagnostic overshadowing causes a delay in autism diagnosis. In particular, a prior diagnosis of ADHD has previously been found to predict later autism diagnosis (e.g., Brett et al., 2016; Miodovnik et al., 2015). By contrast, in our bivariate analysis, earlier‐diagnosed children were more likely than late‐diagnosed children to have an ADHD diagnosis prior to age 7. Also, higher ADHD‐type symptoms at 5 years (SDQ hyperactivity scale), and indeed higher levels of other difficulties (emotional, conduct and peer problems), were associated with earlier diagnosis. Although our data do not directly test this, one possibility is that the contrast between our findings and those of previous studies could reflect recent changes in diagnostic practice. It is possible that an ADHD diagnosis no longer diagnostically overshadows autism, due to increased awareness of autism and growing acceptance of the co‐occurrence of these conditions (Hollingdale, Woodhouse, Young, Fridman, & Mandy, 2020).

Our finding that earlier‐diagnosed children tended to show a decreasing trajectory of EBSDs, whereas later‐diagnosed children show an increasing trajectory replicates recent findings from an Australian population‐representative cohort study (May et al., 2021). Based on both sets of findings, we hypothesise that the relationship between early EBSDs and earlier autism diagnosis involves a child experiencing overt functional difficulties and reduced quality of life, and therefore being more likely to be identified as needing assessment. By contrast, autistic children who have lower levels of co‐occurring EBSDs may present as not experiencing difficulties, and so could fly under the diagnostic radar. Consistent with our hypothesis that co‐occurring difficulties influence an individual’s chance of autism diagnosis comes from our observation that the late‐diagnosed group received their diagnosis at a point when their co‐occurring difficulty trajectories began to equal and exceed those of earlier diagnosed children, that is, after the age of 7 years.

The current study extends the small extant literature using prospective data to chart trajectories of emotional and behavioural problems of autistic people (e.g., Anderson, Maye, & Lord, 2011; Colvert et al., 2021; Gotham, Brunwasser, & Lord, 2015; May et al., 2021; Shattuck et al., 2007; Stringer et al., 2020). Also, our work can help elucidate contradictory findings in previous studies of EBSDs. For example, some have reported an increase in emotional problems for autistic young people over adolescence (Gotham et al., 2015), whereas others have described a decrease (Stringer et al., 2020). Our findings support the idea that emotional difficulties increase in adolescence, at least until 14 years; but also raise the possibility that there are subgroups of autistic young people who have distinct trajectories of these problems. We suggest the value of future work investigating the idea that no single trajectory of EBSDs fits for all autistic young people, but rather that distinct subgroups exist. To this end, work that empirically derives groups of autistic young people based on their EBSD trajectories, using techniques such as growth mixture modelling, will be of value. Increasing understanding of EBSD trajectory groups, including which factors predict membership, can help with targeting appropriate interventions, emphasising prevention, making predictions about future needs and benchmarking change in EBSDs for those receiving interventions.

Children in the late‐diagnosed group experienced a striking increase in social difficulties, as assessed by the SDQ’s Peer Relations scale, between 3 and 14 years of age. In the first two waves of assessment (i.e., 3 and 5 years), the mean score for this group was in the average range but, by the age of 14, their average score was in the abnormal range, and exceeded that of the earlier‐diagnosed group. This increase during childhood and adolescence fits with findings in a different birth cohort, which identified a group of young people who show a marked escalation of autistic social difficulties in late childhood and early adolescence (Mandy, Pellicano, Pourcain, Skuse, & Heron, 2018; Pender, Fearon, St Pourcain, Heron, & Mandy, 2021; Riglin et al., 2021). In these studies, girls are over‐represented amongst those showing this apparent escalation of autistic social difficulties (Mandy et al., 2018; Pender et al., 2021). In our analyses, sex did not moderate the association between diagnostic timing and EBSD change over time: within each group, boys and girls showed similar EBSD trajectories. Nevertheless, we did observe that girls are over‐represented in our late‐diagnosed group, and are thereby over‐represented amongst participants who show a pattern of escalating peer problems. There is currently a diagnostic bias against autistic girls, meaning that they are more likely to be missed (Loomes, Hull, & Mandy, 2017) or diagnosed late (Brett et al., 2016). One possibility, worthy of future investigation, is that autistic girls are more likely than autistic boys to have autism‐related social difficulties that only become overt and significant in adolescence; and that this contributes to the diagnostic bias against females.

The current findings, and those from previous studies (Mandy et al., 2018; Pender et al., 2021; Riglin et al., 2021), raise the possibility that there is a subgroup whose autistic difficulties only become overt and notable in late childhood and early adolescence. It will be important to investigate whether such a group is found in different samples, using well‐validated autism symptom measures. Such work can help uncover the ‘chronogeneity’ of autism, that is, between‐person variability in trajectories of autistic characteristics over time (Georgiades, Bishop, & Frazier, 2017). By better understanding chronogeneity, we can parse the autism phenotype, which is acknowledged to be highly heterogeneous (Abrahams & Geschwind, 2008). This would promote a precision medicine approach to autism clinical care, and likely further research efforts to understand aetiology. In the future, it will be valuable to investigate the extent to which increases in scores on measures of social difficulties are driven by genuine increases in the individual’s level of autistic characteristics or, instead, reflect prior autistic traits having an increasing impact on functioning as demands of the social environment escalate (Mandy et al., 2018).

In the period before their autism was recognised, the late‐diagnosed children in our sample showed lower levels of EBSDs, compared to earlier‐diagnosed children. Does this mean that they did not need an earlier diagnosis, and that their late diagnosis was, in fact, appropriately timed to coincide with the point at which they developed functional impairment? We answer with a qualified ‘no’ to that question, for two reasons. First, many late‐diagnosed children did show clinically severe EBSDs prior to being diagnosed (see Table S2). For example, at age 7 years, a substantial proportion of the late‐diagnosed group scored in the abnormal or borderline range on the SDQ for emotional problems (32%), conduct problems (49%), hyperactivity (51%) and peer problems (50%). Therefore, at least some would likely have benefitted during this time from the support that can come with the recognition of their autism. Second, the late‐diagnosed group went on to develop especially severe difficulties that tended to exceed those of the earlier‐diagnosed children. It is possible that, had they received a timelier diagnosis, their difficulties would have been less likely to escalate in this way (Hosozawa, Sacker, & Cable, 2020).

Our findings must be considered in light of the following limitations. First, like other work based on general‐purpose birth cohort studies, for example, those using The National Cohort of Children’s Health (e.g., Miodovnik et al., 2015), we relied on parent reports to establish the timing of autism diagnosis, without external validation. Nonetheless, parental reports have been shown to have good validity and reliability (Daniels et al., 2012; Sheldrick et al., 2017). Our reliance on parents reporting their child’s autism diagnosis to MCS also meant we could not capture the precise timing of each diagnosis. This constrained our ability to investigate potential differences within our earlier‐ and late‐diagnosed groups. Second, for our measures of EBSDs, we relied on parent reports. Access to self‐report data would have been illuminating, although this would have constrained the age range and ability level of our participants. Third, we could not capture the severity of autistic characteristics or additional co‐occurring difficulties that may present in the autism population including emotion regulation and sleep problems (Lai et al., 2019). Fourth, our reliance on parent reports raises the question of how a child's autism diagnosis may change parents' perception of their child. This could have impacted SDQ scores and the trajectories we described. Fifth, apart from ADHD diagnosis, we did not have records of other mental health diagnoses (e.g., anxiety disorder and conduct disorder) received by participants. Last, we did not have information regarding interventions, in health and education settings, that could have impacted mental health problems, and so cannot assess how these may have influenced the trajectories we observed.

Despite these limitations, we argue our study has value since it adds to the understanding of the characteristics and needs of children who are diagnosed with autism late, after the age of 7 years and, in particular, sheds light on the possible role of timing of co‐occurring EBSDs. Although our study is descriptive – we sought to describe EBSD trajectories for earlier‐ and late‐diagnosed autistic young people – our findings raise the question of what is causing the observed EBSDs and changes in their severity over time. One key question is whether there is any causal association between earlier diagnosis and the subsequent decline in EBSDs we observed. Experimental studies of interventions designed to improve early diagnosis could help address this question, and thereby could inform debates about the value of public health efforts to increase timely diagnosis (e.g., Mandell & Mandy, 2015). Also, to understand any mechanisms potentially linking autism diagnosis with improving EBSDs, researchers should investigate whether environmental changes at home and/or at school that follow diagnosis predict subsequent reductions in ESBDs. In considering the drivers of EBSDs of autistic children, multiple complex processes are likely to be unfolding over time (Cicchetti & Toth, 2009). For example, changes in EBSD might partly reflect changes in core and associated autistic features. This is especially true for our peer problems findings since this SDQ scale has some conceptual overlap with core symptoms of autism, in particular the DSM‐5 diagnostic criterion A3 concerning relationship difficulties (APA, 2013). Longitudinal studies with dimensional measures of autistic and EBSD symptoms will be required for such investigations (e.g., Pickard, Rijsdijk, Happé, & Mandy, 2017). Also, it will be valuable to investigate the impact of transactional processes between the child and their environment, and how these may drive trajectories of various EBSDs (Mandy & Lai, 2016). Further, genetically informed designs will be needed, to characterise the interplay of genetic and environmental risk in the development of EBSDs of autistic people (e.g., Hallett, Ronald, Rijsdijk, & Happé, 2010).

## Ethical standards

The authors assert that all procedures contributing to this work comply with the ethical standards of the relevant national and institutional committees on human experimentation and with the Helsinki Declaration of 1975, as revised in 2008.

**Key points**

- In a nationally‐representative UK birth cohort most (66%) autistic young people received their diagnosis late, only after the age of 8 years.
- These late‐diagnosed children had higher IQ and were more likely to be female, compared to earlier‐diagnosed children.
- Earlier‐diagnosed and late‐diagnosed children had distinct patterns of emotional, behavioural and social difficulties over time.
- Compared to earlier‐diagnosed children, the late‐diagnosed group tended to have milder problems in early childhood, but then showed a steeper growth in their difficulties, such that by 14 years they had higher levels of emotional, behavioural and social difficulties. Research is needed to investigate the extent to which earlier diagnosis has a causal protective effect on psychopathology.
- Many late‐diagnosed children had severe emotional, behavioural and social problems prior to their autism diagnosis, suggesting high levels of unmet clinical need associated with late autism diagnosis.

## Supporting information


**Table S1**. SDQ scores at ages 3, 5, 7, 11 and 14 years by age of autism diagnosis.
**Table S2**. Percentage of children with SDQ scores in normal, borderline abnormal and abnormal ranges at ages 3, 5, 7, 11 and 14 by age of autism diagnosis.
**Table S3**. Sample bias analysis results.
**Table S4**. Emotional problems – unadjusted (Model 1) and adjusted (Model 2) growth models.
**Table S5**. Conduct problems unadjusted (Model 1) and adjusted (Model 2) growth models.
**Table S6**. Hyperactivity unadjusted (Model 1) and adjusted (Model 2) growth models.
**Table S7**. Peer problems – unadjusted (Model 1) and adjusted (Model 2) growth curve models.
