# Supplementary material for: Mental health and social difficulties of late‐diagnosed autistic children, across childhood and adolescence
Source: J Child Psychol Psychiatry. 2022 Feb 16;63(11):1405–14. doi: 10.1111/jcpp.13587 (PMC9790627; doi:10.1111/jcpp.13587)
Supplement: Supplementary file 1 — Table S1. SDQ scores at ages 3, 5, 7, 11 and 14 years by age of autism diagnosis. Table S2. Percentage of children with SDQ scores in normal, borderline abnormal and abnormal ranges at ages 3, 5, 7, 11 and 14 by age of autism diagnosis. Table S3. Sample bias analysis results. Table S4. Emotional problems – unadjusted (Model 1) and adjusted (Model 2) growth models. Table S5. Conduct problems unadjusted (Model 1) and adjusted (Model 2) growth models. Table S6. Hyperactivity unadjusted (Model 1) and adjusted (Model 2) growth models. Table S7. Peer problems – unadjusted (Model 1) and adjusted (Model 2) growth curve models. [file JCPP-63-1405-s001.docx]

**Supporting Information**

**Table S1**

***SDQ scores at ages 3, 5, 7, 11 and 14 years by age of autism diagnosis***

|  | Diagnosed by age 7 years | | Diagnosed between 8 and 14 years | | |
| --- | --- | --- | --- | --- | --- |
|  | *n* | *Mean (SE)* | *N* | *Mean (SE)* | *^a^t* |
| Emotional symptoms |  |  |  |  |  |
| Age 3 | 126 | 1.74 (0.23) | 256 | 1.44 (0.10) | 1.11 |
| Age 5 | 136 | 2.58 (0.28) | 271 | 2.01 (0.12) | 2.44* |
| Age 7 | 136 | 3.72 (0.30) | 268 | 2.43 (0.14) | 4.61*** |
| Age 11 | 140 | 4.25 (0.31) | 275 | 4.06 (0.18) | 0.25 |
| Age 14 | 111 | 3.85 (0.31) | 259 | 4.37 (0.18) | -2.11* |
| Conduct problems |  |  |  |  |  |
| Age 3 | 126 | 3.84 (0.24) | 260 | 3.62 (0.15) | 0.66 |
| Age 5 | 138 | 2.83 (0.18) | 272 | 2.41 (0.11) | 3.07** |
| Age 7 | 136 | 3.17 (0.20) | 268 | 2.51 (0.12) | 3.26** |
| Age 11 | 141 | 2.70 (0.15) | 275 | 2.99 (0.14) | -1.27 |
| Age 14 | 111 | 2.20 (0.23) | 259 | 3.25 (0.18) | -3.53*** |
| Hyperactivity |  |  |  |  |  |
| Age 3 | 127 | 6.17 (0.34) | 253 | 5.26 (0.17) | 4.03*** |
| Age 5 | 137 | 6.58 (0.27) | 271 | 5.29 (0.18) | 4.69*** |
| Age 7 | 136 | 7.41 (0.24) | 268 | 5.59 (0.19) | 6.13*** |
| Age 11 | 141 | 6.71 (0.26) | 275 | 6.16 (0.20) | 2.42* |
| Age 14 | 111 | 5.88 (0.31) | 258 | 6.20 (0.19) | -0.04 |
| Peer relations |  |  |  |  |  |
| Age 3 | 124 | 2.92 (0.20) | 253 | 2.18 (0.15) | 4.09*** |
| Age 5 | 138 | 3.83 (0.26) | 270 | 1.98 (0.14) | 7.62*** |
| Age 7 | 135 | 4.52 (0.25) | 268 | 2.69 (0.18) | 7.40*** |
| Age 11 | 141 | 4.81 (0.23) | 275 | 3.96 (0.17) | 2.38* |
| Age 14 | 111 | 4.65 (0.28) | 259 | 4.79 (0.16) | -0.94 |

*Notes*: **p<.05.**p<.01,***P<.001.* Means are weighted. Ns are unweighted. ^a^*t* test statistic.

**Table S2**

***Percentage of children with SDQ scores in normal, borderline abnormal and abnormal ranges at ages 3, 5, 7, 11 and 14 by age of autism diagnosis***

|  | Diagnosed by age 7 | | | Diagnosed between 8 and 14 | | |  |
| --- | --- | --- | --- | --- | --- | --- | --- |
|  | *Normal* | *Borderline* | *Abnormal* | *Normal* | *Borderline* | *Abnormal* |  |
|  | *%* | *%* | *%* | *%* | *%* | *%* | *F^a^* |
| Emotional symptoms |  |  |  |  |  |  |  |
| Age 3 | 73.88 | 7.70 | 18.42 | 81.13 | 8.05 | 10.82 | 1.85 |
| Age 5 | 64.96 | 9.68 | 25.35 | 77.90 | 7.83 | 14.27 | 3.19** |
| Age 7 | 42.65 | 7.77 | 49.57 | 67.53 | 9.60 | 22.87 | 12.04*** |
| Age 11 | 41.06 | 12.26 | 46.68 | 40.76 | 14.95 | 44.29 | 0.21 |
| Age 14 | 39.20 | 6.43 | 54.36 | 36.39 | 11.21 | 52.39 | 1.04 |
| Conduct problems |  |  |  |  |  |  |  |
| Age 3 | 28.24 | 12.02 | 59.74 | 30.12 | 17.99 | 51.89 | 1.24 |
| Age 5 | 43.91 | 25.95 | 30.14 | 56.78 | 15.99 | 27.24 | 3.01 |
| Age 7 | 39.49 | 18.97 | 41.54 | 51.30 | 17.51 | 31.19 | 2.25 |
| Age 11 | 50.12 | 18.35 | 31.53 | 46.92 | 17.16 | 35.91 | 0.32 |
| Age 14 | 50.08 | 10.96 | 38.96 | 41.48 | 15.43 | 43.10 | 1.00 |
| Hyperactivity problems |  |  |  |  |  |  |  |
| Age 3 | 36.67 | 8.25 | 55.08 | 47.04 | 15.25 | 37.71 | 4.50* |
| Age 5 | 33.93 | 10.69 | 55.38 | 51.88 | 13.75 | 34.38 | 6.33** |
| Age 7 | 21.47 | 8.70 | 69.83 | 48.35 | 7.66 | 44.00 | 12.20*** |
| Age 11 | 32.27 | 7.24 | 60.48 | 37.32 | 11.67 | 51.01 | 1.51 |
| Age 14 | 31.24 | 9.17 | 59.59 | 36.08 | 8.60 | 55.31 | 0.39 |
| Peer relations problems |  |  |  |  |  |  |  |
| Age 3 | 40.01 | 14.51 | 45.48 | 59.70 | 12.94 | 27.37 | 7.70*** |
| Age 5 | 29.65 | 16.92 | 53.43 | 63.17 | 13.85 | 22.98 | 18.01*** |
| Age 7 | 15.37 | 15.14 | 69.49 | 50.06 | 12.87 | 37.06 | 17.86*** |
| Age 11 | 16.96 | 11.16 | 71.88 | 30.50 | 14.16 | 55.35 | 4.16* |
| Age 14 | 18.69 | 5.41 | 75.89 | 19.06 | 8.62 | 72.33 | 0.59 |

*Notes*: **p<.05.* Percentages are weighted.

*^a^*F statistic for design-based Pearson chi-square that is converted to F test to account for the MCS sampling design

**Table S3**

**Sample bias analysis results**

|  | Analytic sample | | Non-analytic sample | | |  |  |  |
| --- | --- | --- | --- | --- | --- | --- | --- | --- |
| Categorical variables | *Freq.* | *%* | *Freq. % ^a^F* | | |  | | |
| Child is female | 34 | 24.27 | 95 | 22.43 | 0.14 |  |  |  |
| Child has white ethnicity | 131 | 91.60 | 383 | 91.86 | 0.01 |  |  |  |
| ADHD Diagnosis | | |  |  | |  |  |  |
| No diagnosis | 108 | 72.98 | 292 | 70.57 | 2.18 |  |  |  |
| Late diagnosis | 16 | 8.97 | 79 | 17.25 |  |  |  |  |
| Early diagnosis | 24 | 18.05 | 59 | 12.17 |  |  |  |  |
| Mother has university degree (by age 14) | 22 | 14.92 | 71 | 20.41 | 1.68 |  |  |  |
| Continuous variables | *n(valid)* | *Mean (SE)* | *n(valid)* | *Mean (SE)* | *^c^t* |  |  |  |
| Family socio-economic disadvantage (age 5) | 120 | 0.25(0.03) | 419 | 0.25(0.02) | 0.87 |  |  |  |
| Mother’s age at birth of child | 147 | 24.23(0.59) | 426 | 24.92(0.32) | -1.61 |  |  |  |
| Child general cognitive ability (age 5) | 98 | 94.10(1.72) | 361 | 94.63(0.99) | -1.42 |  |  |  |

*Notes*: ***p<.01.***p<.001.* Means and percentages are weighted. Ns are unweighted. The analytic sample comprises singleton and first-born twins/triplets, those with valid diagnosis information in at least three sweeps and a stable autism diagnosis. The non-analytic sample comprises singleton and first-born twins-triplets with a report of an autism diagnosis in at least one wave (age 3, 5, 7, 11 or 14) but either fewer than 3 valid waves of information on diagnosis or an unstable diagnosis.

^a^ *F* statistic for design-based Pearson chi-square that is converted to F test to account for the MCS sampling design.

^b^ *t* test statistic.

**Table S4**

**Emotional problems - unadjusted (Model 1) and adjusted (Model 2) growth models**

|  |  | | | **Model 1** |  | |  | | **Model 2** | |  |
| --- | --- | --- | --- | --- | --- | --- | --- | --- | --- | --- | --- |
| **Fixed effects** | **Coeff.** | | | **SE** | **95% CI** | | **Coeff.** | | **SE** | | **95% CI** |
| Constant | 2.427*** | | | 0.185 | [2.064,2.789] | | 4.300*** | | 0.646 | | [3.035,5.567] |
| Age | 0.321*** | | | 0.032 | [0.258,0.383] | | 0.216* | | 0.106 | | [0.009,0.423] |
| Age^2^ | -0.015*** | | | 0.003 | [-0.021,-0.009] | | -0.015*** | | 0.003 | | [-0.021,-0.009] |
| Late autism diagnosis | -0.610** | | | 0.167 | [-0.938,-0.281] | | -0.507** | | 0.172 | | [-0.845,-0.169] |
| Late autism diagnosis x age | 0.084** | | | 0.029 | [0.028,0.140] | | 0.076** | | 0.030 | | [0.019,0.135] |
| Late ADHD diagnosis |  | | |  |  | | 1.365*** | | 0.306 | | [-0.765,1.966] |
| Late ADHD diagnosis x age |  | | |  |  | | -0.015 | | 0.043 | | [-0.100,0.069] |
| No ADHD diagnosis |  | | |  |  | | -0.117 | | 0.238 | | [-0.583,0.348] |
| No ADHD diagnosis x age |  | | |  |  | | -0.069 | | 0.040 | | [-0.148,0.011] |
| Cognitive ability |  | | |  |  | | -0.013* | | 0.005 | | [-0.023,-0.003] |
| Cognitive ability x age |  | | |  |  | | 0.0003 | | 0.001 | | [-0.002,0.002] |
| Female |  | | |  |  | | -0.126** | | 0.194 | | [-0.506,0.254] |
| Female x age |  | | |  |  | | -0.082 | | 0.033 | | [-0.018,0.147] |
| Maternal education |  | | |  |  | | -0.011 | | 0.215 | | [-0.431,0.410] |
| Maternal education x age |  | | |  |  | | -0.064 | | 0.037 | | [-0.135,0.008] |
| Maternal age of first birth |  | | |  |  | | -0.017 | | 0.016 | | [-0.017,0.014] |
| Maternal age of first birth x age |  | | |  |  | | 0.003 | | 0.003 | | [-0.002,0.008] |
| **Random effects (variances)** | |  |  | | |  | |  | |  | |
| Between-child intercept | 1.763* | | | 0.171 | [1.458,2.132] | | 1.691* | | 0.167 | | [1.395,2.052] |
| Between-child slope | 0.039* | | | 0.005 | [0.030,0.051] | | 0.037* | | 0.005 | | [0.029,0.048] |
| Between-occasion | 2.404* | | | 0.098 | [2.220,2.603] | | 2.404* | | 0.098 | | [2.220,2.603] |

*Note*. ^*^*p*<.05; ^**^*p* < .01; **p*<.001 Age of diagnosis reference group = Diagnosed by age 7. Age of ADHD diagnosis reference group = early diagnosis. Age is mean centred at around 5 years of age. All estimates have been adjusted for the MCS area strata

**Table S5**

**Conduct problems unadjusted (Model 1) and adjusted (Model 2) growth models**

|  | |  | | **Model 1** |  | |  | | **Model 2** | |  |
| --- | --- | --- | --- | --- | --- | --- | --- | --- | --- | --- | --- |
| **Fixed effects** | | **Coeff.** | | **SE** | **95% CI** | | **Coeff.** | | **SE** | | **95% CI** |
| Constant | | 3.170*** | | 0.185 | [2.808,3.532] | | 6.147*** | | 0.575 | | [5.020,7.273] |
| Age | | -0.272*** | | 0.028 | [-0.326,-0.218] | | -0.354*** | | 0.087 | | [-0.524,-0.183] |
| Age^2^ | | 0.023*** | | 0.003 | [0.017,0.028] | | 0.022*** | | 0.003 | | [0.017,0.028] |
| Late autism diagnosis | | -0.438** | | 0.167 | [-0.765,-0.110] | | -0.359* | | 0.155 | | [-0.663,-0.054] |
| Late autism diagnosis x age | | 0.109*** | | 0.024 | [0.062,0.155] | | 0.099*** | | 0.024 | | [0.052,0.146] |
| Late ADHD diagnosis | |  | |  |  | | -1.006*** | | 0.257 | | [-1.510,-0.502] |
| Late ADHD diagnosis x age | |  | |  |  | | 0.065 | | 0.039 | | [-0.011,0.142] |
| No ADHD diagnosis | |  | |  |  | | -1.691*** | | 0.215 | | [-2.112,-1.270] |
| No ADHD diagnosis x age | |  | |  |  | | -0.024 | | 0.033 | | [-0.088,0.041] |
| Cognitive ability | |  | |  |  | | 0.002 | | 0.005 | | [-0.007,0.011] |
| Cognitive ability x age | |  | |  |  | | 0.0001 | | 0.001 | | [-0.001,0.002] |
| Female | |  | |  |  | | -0.131** | | 0.175 | | [-0.474,0.212] |
| Female x age | |  | |  |  | | 0.007 | | 0.027 | | [-0.046,0.059] |
| Maternal education | |  | |  |  | | -0.168 | | 0.194 | | [-0.548,0.211] |
| Maternal education x age | |  | |  |  | | -0.071* | | 0.030 | | [-0.129,-0.013] |
| Maternal age of first birth | |  | |  |  | | -0.060*** | | 0.014 | | [-0.088,-0.032] |
| Maternal age of first birth x age | |  | |  |  | | 0.002 | | 0.002 | | [-0.002,0.006] |
| **Random effects (variances)** |  | |  | | |  | |  | |  | |
| Between-child intercept | | 1.383* | | 0.133 | [1.145,1.671] | | 1.383* | | 0.133 | | [1.145,1.671] |
| Between-child slope | | 0.019* | | 0.003 | [0.013,0.026] | | 0.019* | | 0.003 | | [0.013,0.026] |
| Between-occasion | | 1.992* | | 0.082 | [1.838,2.159] | | 1.992* | | 0.082 | | [1.838,2.159] |

*Note*. ^*^*p*<.05; ^**^*p* < .01; **p*<.001 Age of autism diagnosis reference group = Diagnosed by age 7. Age of ADHD diagnosis reference group = early diagnosis. Age is mean centred at around 5 years of age. All estimates have been adjusted for the MCS area strata.

**Table S6**

**Hyperactivity unadjusted (Model 1) and adjusted (Model 2) growth models**

|  |  | | | **Model 1** |  | |  | | **Model 2** | |  |
| --- | --- | --- | --- | --- | --- | --- | --- | --- | --- | --- | --- |
| **Fixed effects** | **Coeff.** | | | **SE** | **95% CI** | | **Coeff.** | | **SE** | | **95% CI** |
| Constant | 6.791*** | | | 0.257 | [6.287,7.296] | | 11.208*** | | 0.797 | | [9.645,12.771] |
| Age | 0.110** | | | 0.033 | [0.045,0.175] | | 0.110 | | 0.103 | | [-0.091,0.311] |
| Age^2^ | -0.018*** | | | 0.003 | [-0.024,-0.011] | | -0.018*** | | 0.004 | | [-0.024,-0.011] |
| Late diagnosis | -1.331*** | | | 0.232 | [-1.785,-0.878] | | -1.149*** | | 0.214 | | [-1.568,-0.730] |
| Late diagnosis x age | 0.111*** | | | 0.028 | [0.055,0.166] | | 0.111*** | | 0.028 | | [0.055,0.166] |
| Late ADHD diagnosis |  | | |  |  | | -1.230*** | | 0.353 | | [-1.922,-0.538] |
| Late ADHD diagnosis x age |  | | |  |  | | 0.058 | | 0.046 | | [-0.032,0.149] |
| No ADHD diagnosis |  | | |  |  | | -2.462*** | | 0.295 | | [-3.040,-1.885] |
| No ADHD diagnosis x age |  | | |  |  | | -0.039 | | 0.039 | | [-0.115,0.037] |
| Cognitive ability |  | | |  |  | | -0.037*** | | 0.007 | | [-0.050,-0.024] |
| Cognitive ability x age |  | | |  |  | | 0.001 | | 0.001 | | [-0.001,0.002] |
| Female |  | | |  |  | | -0.547* | | 0.266 | | [-1.068,-0.026] |
| Female x age |  | | |  |  | | -0.084* | | 0.033 | | [-0.148,-0.019] |
| Maternal education |  | | |  |  | | -0.155 | | 0.266 | | [-0.677,0.367] |
| Maternal education x age |  | | |  |  | | -0.073* | | 0.035 | | [-0.140,-0.015] |
| Maternal age of first birth |  | | |  |  | | -0.029 | | 0.019 | | [-0.067,0.008] |
| Maternal age of first birth x age |  | | |  |  | | 0.002 | | 0.002 | | [-0.003,0.007] |
| **Random effects (variances)** | |  |  | | |  | |  | |  | |
| Between-child intercept | 4.047* | | | 0.331 | [3.447,4.751] | | 2.922* | | 0.255 | | [2.463,3.468] |
| Between-child slope | 0.031* | | | 0.005 | [0.022,0.043] | | 0.025* | | 0.005 | | [0.018,0.036] |
| Between-occasion | 2.770* | | | 0.116 | [2.553,3.006] | | 2.798* | | 0.121 | | [2.578,3.038] |

*Note*. ^*^*p*<.05; ^**^*p* < .01; **p*<.001 Age of diagnosis reference group = Diagnosed by age 7. Age of ADHD diagnosis reference group = early diagnosis. Age is mean centred at around 5 years of age. All estimates have been adjusted for the MCS area strata.

**Table S7**

**Peer problems - unadjusted (Model 1) and adjusted (Model 2) growth curve models**

|  |  | | | **Model 1** |  | |  | | **Model 2** | |  |
| --- | --- | --- | --- | --- | --- | --- | --- | --- | --- | --- | --- |
| **Fixed effects** | **Coeff.** | | | **SE** | **95% CI** | | **Coeff.** | | **SE** | | **95% CI** |
| Constant | 4.300*** | | | 0.646 | [3.035,5.567] | | 4.921*** | | 0.668 | | [3.612,6.229] |
| Age | 0.120*** | | | 0.033 | [0.055,0.184] | | 0.033 | | 0.107 | | [-0.176,0.242] |
| Age^2^ | 0.002*** | | | 0.003 | [-0.005,0.009] | | 0.002*** | | 0.003 | | [-0.005,0.009] |
| Late autism diagnosis | -1.410*** | | | 0.169 | [-1.742,-1.078] | | -1.357*** | | 0.176 | | [-1.702,-1.011] |
| Late autism diagnosis x age | 0.135*** | | | 0.029 | [0.080,0.192] | | 0.130*** | | 0.030 | | [0.072,0.119] |
| Late ADHD diagnosis |  | | |  |  | | 0.155 | | 0.291 | | [-0.415,0.725] |
| Late ADHD diagnosis x age |  | | |  |  | | -0.073 | | 0.049 | | [-0.168,0.023] |
| No ADHD diagnosis |  | | |  |  | | 0.049 | | 0.243 | | [-0.427,0.524] |
| No ADHD diagnosis x age |  | | |  |  | | -0.065 | | 0.041 | | [-0.145,0.015] |
| Cognitive ability |  | | |  |  | | -0.010* | | 0.005 | | [-0.021,0.001] |
| Cognitive ability x age |  | | |  |  | | 0.001 | | 0.001 | | [-0.001,0.003] |
| Female |  | | |  |  | | -0.100 | | 0.198 | | [-0.488,0.287] |
| Female x age |  | | |  |  | | 0.036 | | 0.033 | | [-0.029,0.101] |
| Maternal education |  | | |  |  | | 0.105 | | 0.219 | | [-0.324,0.533] |
| Maternal education x age |  | | |  |  | | 0.001 | | 0.037 | | [-0.070,0.073] |
| Maternal age of first birth |  | | |  |  | | -0.012 | | 0.016 | | [-0.044,0.019] |
| Maternal age of first birth x age |  | | |  |  | | -0.0004 | | 0.003 | | [-0.006,0.005] |
| **Random effects (variances)** | |  |  | | |  | |  | |  | |
| Between-child intercept | 1.383* | | | 0.133 | [1.394,2.072] | | 1.667* | | 0.170 | | [1.366,2.035] |
| Between-child slope | 0.033* | | | 0.005 | [0.025,0.045] | | 0.033* | | 0.005 | | [0.024,0.044] |
| Between-occasion | 2.736* | | | 0.112 | [2.525,2.966] | | 2.737* | | 0.112 | | [2.526,2.966] |

*Note*. ^*^*p*<.05; ^**^*p* < .01; **p*<.001 Age of diagnosis reference group = Diagnosed by age 7. Age of ADHD diagnosis reference group = early diagnosis. Age is mean centred at around 5 years of age. All estimates have been adjusted for the MCS area strata.
